# Protocol for live imaging of axonal transport in iPSC-derived iNeurons

**DOI:** 10.1016/j.xpro.2024.103556

**Published:** 2025-01-12

**Authors:** Dan Dou, Erika L.F. Holzbaur, C. Alexander Boecker

**Affiliations:** 1 Department of Physiology, Perelman School of Medicine, University of Pennsylvania, Philadelphia, PA 19104, USA; 2 Neuroscience Graduate Group, University of Pennsylvania Perelman School of Medicine, Philadelphia, PA 19104, USA; 3 Department of Neurology, University Medical Center Goettingen, 37077 Goettingen, Germany

**Keywords:** cell biology, microscopy, stem cells

## Abstract

Studies of human induced pluripotent stem cell (iPSC)-derived neurons promise important insights into neurodegenerative diseases. Here, we present a protocol for live imaging of axonal transport in glutamatergic iPSC-derived neurons (iNeurons). We describe steps for the differentiation of iPSCs into iNeurons via PiggyBac-mediated neurogenin 2 (NGN2) delivery, iNeuron culture and transfection, and the acquisition and analysis of time-lapse images. Our protocol is optimized for the widely available catalog of KOLF2.1J iPSCs with mutations relevant to neurodegenerative diseases but is also applicable to other iPSC lines.

For complete details on the use and execution of this protocol, please refer to Dou et al.^1^^,^^2^

## Highlights

• Protocol to enable live imaging of axonal transport in human iPSC-derived iNeurons
• Differentiation of iPSCs into iNeurons using PiggyBac-mediated NGN2 delivery
• Culture, transfection, and live imaging of fluorescent axonal cargo in iNeurons
• All steps optimized for use in KOLF2.1J iPSCs

## Abstract (editor highlights)

Publisher’s note: Undertaking any experimental protocol requires adherence to local institutional guidelines for laboratory safety and ethics.

## Graphical abstract

**Figure:**
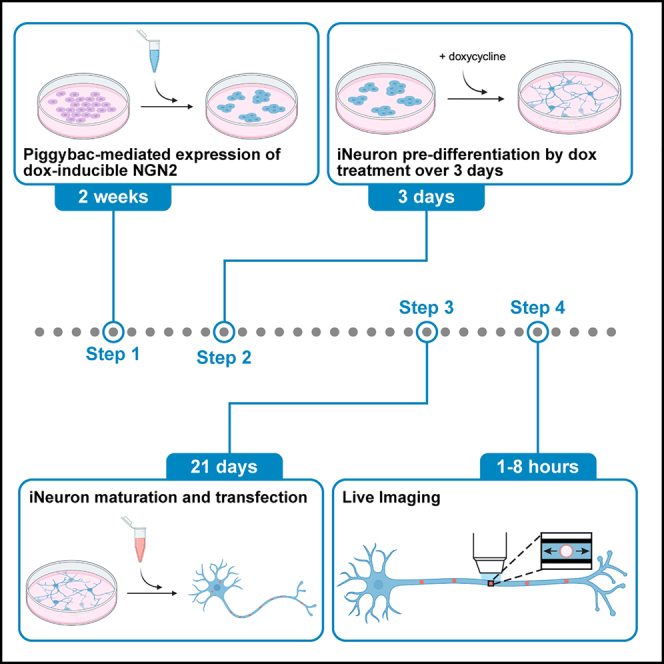

## Before you begin

Overexpression of NGN2 in human pluripotent stem cells allows for reproducible generation of excitatory glutamatergic neurons.^3^ Advancements in NGN2-based differentiation protocols^4^ and characterization of induced pluripotent stem cell (iPSC) lines^5^^,^^6^ have made this approach more accessible than ever as a powerful tool for modeling of human neuronal physiology and pathophysiology. The following protocol enables live imaging of axonal transport in iPSC-derived glutamatergic iNeurons. We describe the use of a PiggyBac delivery system to stably express doxycycline-inducible NGN2 in iPSCs, subsequent differentiation of these iPSCs into iNeurons, and the culture and transfection of iNeurons for live imaging experiments. The protocol has been optimized for iPSCs from the KOLF2.1J parental line, which have been used by the iPSC Neurodegenerative Initiative (iNDI) to generate a widely available catalog of iPSCs with knock-in of various mutations relevant to neurodegenerative disorders.^5^ However, it is also applicable to other iPSC lines.

Perform all cell culture work in a biosafety cabinet using sterile technique. Culture cells in a 37°C humidified incubator with 5% CO_2_.

***Note:*** iPSCs should be tested to rule out mycoplasma contamination, and cytogenetic analysis of G-banded metaphase cells should be performed to confirm a normal karyotype.

### Preparations for iPSC culture

**Timing: 2 days**

**Timing: 20 min (for step 1)**

**Timing: 2 days (for step 2)**

**Timing: 90 min (for step 3)**

**Timing: 2 days (for step 4)**

Take the following steps in preparation for “cell culture of undifferentiated iPSCs.”

1. Prepare ROCK inhibitor Stock Solution (10 mM, 1000x).

***Note:*** This step can be done months in advance. Aliquot to minimize freeze-thaw cycles when possible.

2. Prepare aliquots of Matrigel Growth Factor Reduced (GFR).
  a. Thaw bottle of Matrigel overnight (12–16 h) by placing it in a styrofoam container filled with ice within a refrigerator.
  b. On the next day, label autoclaved 1.5 mL Eppendorf tubes and chill by placing on ice.
  c. Chill P1000 pipette tip by up-and-down pipetting of ice-cold DMEM/F-12 media several times.
  d. Use chilled pipette tip to prepare 200 μL aliquots of Matrigel stock.
  e. Re-freeze and store Matrigel aliquots at −80°C. These individual aliquots should not be re-frozen after use.

**CRITICAL:** Matrigel polymerizes rapidly at room temperature (RT; 18°C–22°C). Work on ice and avoid touching the bottom of bottles/tubes where Matrigel is located.

***Note:*** Matrigel aliquoting can be done months in advance.

3. Coat cell culture dishes with Matrigel.
  a. Remove Matrigel aliquot from the −80°C freezer and place on ice.
  b. Dilute 200 μL of Matrigel stock aliquot 1:100 with 20 mL ice-cold DMEM/F-12 (Gibco #11330033).
    i. Aliquot 20 mL of DMEM/F-12 to a conical tube and place on ice.
    ii. Add a small volume of the aliquoted, ice-cold DMEM/F-12 (e.g., 750 μL) to the Eppendorf tube containing the frozen Matrigel stock aliquot and thaw Matrigel by pipetting up and down several times.
    iii. Transfer DMEM/F-12 + thawed Matrigel back to conical tube and mix by pipetting up and down several times with a 10 mL serological pipette.
  c. Add 5 mL of diluted Matrigel per 10 cm dish, 10 mL of diluted Matrigel per 15 cm dish, or 1.5 mL of diluted Matrigel per well of a 6-well plate. Coat by placing in 37°C incubator for 1 h.

***Note:*** If one Matrigel stock aliquot does not provide enough diluted Matrigel to coat all dishes required for a particular experiment, scale up this step. For example, dilute two Matrigel stock aliquots (400 μL) with 40 mL of ice-cold DMEM/F-12.

***Note:*** The formulation of DMEM/F-12 media used for diluting Matrigel (Gibco #11330033) is different from the formulation used to prepare Induction Media (Gibco #1133032).

**Pause point:** Instead of coating at 37°C for one hour, dishes not to be used immediately can be wrapped with Parafilm M Lab Film and stored on a level surface in a 4°C fridge for up to 2 weeks. When ready to use, remove dishes from fridge and allow to return to RT (18°C–22°C) in a cell culture hood before aspirating Matrigel solution and plating cells.

4. Prepare complete Essential 8 media.
  a. Thaw the frozen Essential 8 supplement at RT (18°C–22°C) or at 4°C overnight (12–16 h).
    **CRITICAL:** Do not thaw the frozen Essential 8 supplement at 37°C.
  b. Transfer Essential 8 supplement to the bottle of Essential 8 basal media.
  c. Swirl the Essential 8 bottle to mix.
    ***Note:*** Store complete Essential 8 media at 4°C for up to two weeks. For long-term storage, aliquot 45 mL of complete Essential 8 media into 50 mL conical tubes and store at −20°C. When ready to use, thaw aliquots overnight (12–16 h) at 4°C.
    **CRITICAL:** Do not warm complete Essential 8 media at 37°C, as this can rapidly decrease the activity of cytokines in Essential 8 media. Before use, warm complete Essential 8 media at RT (18°C–22°C) until no longer cold to the touch.

### Cell culture of undifferentiated iPSCs

**Timing: 3–4 days**

**Timing: 30 min (for step 5)**

**Timing: 2–3 days (for steps 6a and b)**

**Timing: 15 min (for step 6c)**

The following steps describe thawing, culturing, and maintenance passaging of iPSCs. Take these steps in preparation for “PiggyBac-mediated transfection for the stable expression of NGN2” and “differentiation of iPSCs into iNeurons.”

***Note:*** Prior to starting the following steps, review and carry out the steps outlined in “preparations for iPSC culture.” In particular, have Matrigel-coated dishes ready for use, either in the incubator for same-day use or moved from the refrigerator to the RT (18°C–22°C) tissue culture hood.

5. Thaw iPSCs and plate onto a Matrigel-coated 10 cm dish.
  ***Note:*** For “PiggyBac-mediated transfection for the stable expression of NGN2,” these iPSCs should be wild-type (WT) KOLF2.1J iPSCs. For “differentiation of iPSCs into iNeurons,” these iPSCs should be stably expressing doxycycline-inducible NGN2.
  a. Transport a cryovial containing iPSCs on ice from liquid nitrogen storage.
  b. For best viability, quickly thaw vials in a 37°C water bath.
  c. Move vial to tissue culture hood when only a small amount of ice remains visible.
  d. Transfer cells to a 15 mL conical tube.
    ***Note:*** Minimizing flow of cell suspension down the side of the tube may improve cell viability.
  e. Add 3 mL Essential 8 media supplemented with 10 μM ROCK inhibitor (RI).
  f. Centrifuge tube at 200 g for 4 min.
  g. Aspirate supernatant and resuspend cell pellet in 2 mL Essential 8 + RI by gently pipetting up and down with a P1000 pipette.
  h. Perform live cell count using Trypan blue.
  i. Transfer 1 million iPSCs to a new 15 mL tube and add Essential 8 + RI to a total volume of 10 mL.
  j. Mix by pipetting up and down gently 5x with a 10 mL serological pipette until cell suspension appears homogeneous.
  k. Remove excess Matrigel solution from 10 cm dish and, without allowing the surface to dry, gently add iPSCs (in 10 mL Essential 8 + RI) to dish.
  l. Slide dish front-to-back and side-to-side several times to evenly distribute cells.
    ***Note:*** These movements should be forceful enough to allow for spreading of cells, but gentle enough to prevent splashing of media out of the dish or onto the lid.
  m. Place dish in 37°C incubator and again move front-to-back and side-to-side several times to evenly distribute cells.
6. Culture iPSCs and split using ReLeSR reagent when reaching 70–80% confluency.
  a. The day after thawing, aspirate all Essential 8 + RI and replace with 10 mL Essential 8 without RI.
  b. Continue feeding iPSCs daily with fresh Essential 8 until cells reach 70–80% confluency.
    ***Note:*** This should typically occur 3 days after thawing. Visually assess cultures daily using a tissue culture microscope to monitor growth. Healthy iPSCs should grow in colonies with tightly clustered cells and clearly defined borders. Watch out for spontaneous differentiation with loss of border integrity and loose cellular packing. A low amount of spontaneous differentiation is normal, but higher amounts indicate poor cell quality.
  c. Split cells 1:6 onto a 10 cm dish coated with Matrigel using ReLeSR passaging reagent.
    i. Aspirate culture media and rinse cells with 5 mL PBS (without Ca^2+^ and Mg^2+^).
    ii. Add 3 mL of ReLeSR reagent and incubate for 1 min at RT (18°C–22°C).
    iii. Aspirate ReLeSR so that iPSCs are exposed to only a thin film of liquid.
    iv. Incubate for 8 min at RT (18°C–22°C).
    v. Add 6 mL Essential 8 (without RI) and detach colonies by holding the dish with one hand and tapping the side of the dish with the other hand for 30–60 s.
    vi. Transfer cell suspension to a 15 mL conical tube and pipette up and down 5x with a 5 mL serological pipette to break up larger cell aggregates.
    vii. Split cells 1:6 onto a new 10 cm dish: transfer 1 mL cell suspension to a 15 mL conical tube and add Essential 8 (without RI) to a total volume of 10 mL.
    viii. Remove Matrigel solution from 10 cm dish and directly add 10 mL of diluted iPSCs.
    ix. Check cells under the microscope.
      ***Note:*** Small cell aggregates with a size of approximately 50–200 μm (∼10 cells) should be visible.
    x. Place the dishes in a 37°C incubator and move front-to-back and side-to-side several times to evenly distribute cell aggregates.

### Preparations for the differentiation of iPSCs into iNeurons

**Timing: 1–2 h**

Take the following steps in preparation for “differentiation of iPSCs into iNeurons”.

7. Prepare Doxycycline Stock Solution (2 mg/mL, 1000x).

***Note:*** This can be done months in advance. Aliquot to minimize freeze-thaw cycles when possible.

8. Prepare BDNF (10 μg/mL, 1000x), NT-3 (10 μg/mL, 1000x), and Laminin (1 mg/mL, 1000x) Stock Solutions.

***Note:*** This can be done months in advance. Once thawed, store individual aliquots at 4°C for up to 2 weeks to avoid freeze-thaw cycles.

9. Prepare Induction Media.

***Note:*** Induction Media (without doxycycline and ROCK inhibitor) can be stored at 4°C for 2–3 months. Add doxycycline and ROCK inhibitor fresh on the day of use.

***Note:*** The formulation of DMEM/F-12 media used for preparing Induction Media (Gibco #11330032) is different from the formulation used to dilute Matrigel (Gibco #1133033).

10. Prepare iNeuron Freezing Media on the day of use.

### Preparations for the culture of iNeurons

**Timing: 2 days**

Take the following steps in preparation for “cell culture of iNeurons”.

11. Prepare BDNF (10 μg/mL, 1000x), NT-3 (10 μg/mL, 1000x), and Laminin (1 mg/mL, 1000x) Stock Solutions.

***Note:*** This can be done months in advance. Once thawed, store individual aliquots at 4°C for up to 2 weeks to avoid freeze-thaw cycles.

12. Prepare 5-Fluoro-2′-deoxyuridine (FdU, 10 mM, 1000x) and uridine (10 mM, 1000x) Stock Solutions.

***Note:*** This can be done months in advance. Aliquot to minimize freeze-thaw cycles when possible.

13. Prepare Poly-L-ornithine (PLO) Stock Solution (1 mg/mL, 10x).
  a. Suspend 50 mg PLO in 50 mL 0.1 M borate buffer.
  b. After aliquoting, store 10x PLO stock at −80°C. These individual aliquots should not be re-frozen after use.

***Note:*** PLO Stock Solution can be prepared months in advance.

14. On the day prior to plating (Culture Day −1): coat imaging dishes.
  a. Dilute 10x PLO stock in ddH_2_O to 1x solution.
  b. In the cell culture hood, coat 35 mm glass bottom imaging dishes with 1 mL of 1x PLO solution.
    ***Note:*** This will comfortably cover the glass center of the imaging dish. It is not necessary to coat the plastic ring surrounding the glass center.
  c. Move dishes to sterile 37°C cell culture incubator overnight (12–16 h).
15. Prepare iNeuron Culture Media fresh on the day of use.
16. Prepare Imaging Media fresh on day of live imaging.

## Key resources table

**Table:** 

REAGENT or RESOURCE	SOURCE	IDENTIFIER
**Chemicals, peptides, and recombinant proteins**		

Matrigel growth factor reduced	Corning	Cat# 354230
Essential 8 medium	Thermo Fisher Scientific	Cat# A1517001
PBS, pH 7.4 (without calcium chloride, without magnesium chloride)	Thermo Fisher Scientific	Cat# 10010049
ReLeSR	STEMCELL Technologies	Cat# 05872
Accutase	Sigma-Aldrich	Cat# A6964
ROCK inhibitor Y-27632	Selleckchem	Cat# S1049
Tet System approved FBS	Takara	Cat# 631107
Knockout serum replacement (KOSR)	Thermo Fisher Scientific	Cat# 10828010
Puromycin dihydrochloride	Research Products International	Cat# P33020
DMEM/F-12	Thermo Fisher Scientific	Cat# 11330033
DMEM/F-12, HEPES	Thermo Fisher Scientific	Cat# 11330032
N2 supplement	Thermo Fisher Scientific	Cat# 17502048
Non-essential amino acids (NEAA)	Thermo Fisher Scientific	Cat# 11140050
GlutaMAX, 100x	Thermo Fisher Scientific	Cat# 35050061
Boric acid	Carl Roth	Cat# 6943.1
Doxycycline	Sigma-Aldrich	Cat# D9891
Poly-L-ornithine	Sigma-Aldrich	Cat# P3655
BrainPhys Neuronal medium	STEMCELL Technologies	Cat# 05790
Laminin	Corning	Cat# 354232
BDNF	PeproTech	Cat# 450-02
NT-3	PeproTech	Cat# 450-03
IgG- and protease-free BSA	Carl Roth	Cat# 3737.2
B27 supplement	Thermo Fisher Scientific	Cat# 17504-044
Hibernate A Low-Fluorescence medium	BrainBits	Cat# NC0442869
Opti-MEM medium	Thermo Fisher Scientific	Cat# 31985062
Lipofectamine Stem Transfection Reagent	Thermo Fisher Scientific	Cat# STEM00003
5-Fluoro-2′-deoxyuridine	Sigma-Aldrich	Cat# F0503
Uridine	Sigma-Aldrich	Cat# U3003

**Critical commercial assays**		

Plasmid Maxi Kit	QIAGEN	Cat#12163

**Experimental models: Cell lines**		

Human: KOLF2.1J iPSCs	B. Skarnes (Jackson Laboratory, Connecticut)	RRID: CVCL_B5P3

**Recombinant DNA**		

Plasmid: PB-TO-hNGN2	Gift from iPSC Neurodegenerative Disease Initiative (iNDI) & Michael Ward	Addgene plasmid #172115
Plasmid: piggyBac transposase vector	Transposagen	N/A
Plasmid: PGK EGFP-LC3B	Dou et al., 2023^1^	Addgene plasmid #200427
Plasmid: PGK mScarlet-LC3B	Dou et al., 2023^1^	Addgene plasmid #200083
Plasmid: PGK 4xMito-mEmerald	Dou et al., 2023^1^	Addgene plasmid #200430
Plasmid: PGK mScarlet-Synaptophysin	Dou et al., 2024^2^	Addgene plasmid #206145

**Software and algorithms**		

Fiji (Release 2.9.0)	NIH, USA	http://fiji.sc, RRID:SCR_002285
Matlab R2022a	MathWorks	https://www.mathworks.com/products/matlab.html, RRID:SCR_001622
KymoSuite (custom MATLAB script)	Guedes-Dias et al., 2019	https://github.com/jnirschl/kinesin-3_guedes-dias_2018/tree/master/kymoSuite, https://zenodo.org/record/2530934
Volocity	PerkinElmer	https://www.perkinelmer.com, RRID:SCR_002668
VisiView 5.0.0.24	Visitron	https://www.visitron.de/products/visiviewr-software.html, RRID:SCR_022546
Adobe Illustrator 2022	Adobe	https://www.adobe.com/products/illustrator.html, RRID:SCR_010279
BioRender	BioRender	https://biorender.com/, RRID:SCR_018361

**Other**		

35 mm #1.5 glass bottom imaging dishes	MatTek	Cat# P35G-1.5-20-C
Parafilm M Lab film	Sigma-Aldrich	Cat# HS234526A
Trypan blue solution, 0.4% (with countess cell counting chamber slides)	Invitrogen	Cat# T10282
Perkin Elmer UltraView VoX Spinning disk confocal For details, see “microscopy equipment” in the materials and equipment section	PerkinElmer	N/A
Nikon immersion oil, Type F	Nikon	Cat# MXA22168

## Materials and equipment

**Table:** ROCK inhibitor Stock Solution (10 mM, 1000x)

Reagent	Final concentration	Amount
ROCK inhibitor Y-27632	10 mM	10 mg
PBS, pH 7.4 (without calcium chloride, without magnesium chloride)	N/A	3.12 mL
**Total**	**10 mM**	**3.12 mL**

Store as 50 μL aliquots for up to one year.

**Table:** iPSC Freezing Media

Reagent	Final concentration	Amount
Essential 8	70%	14 mL
Knockout Serum Replacement (KOSR)	20%	4 mL
DMSO	10%	2 mL
ROCK inhibitor	10 μM	20 μL
**Total**	**N/A**	**20 mL**

Prepare fresh on the day of use. Adjust volumes as needed to freeze down all iPSCs.

***Alternatives:*** Doxycycline- and tetracycline-free FBS can be used instead of knockout serum replacement.

**CRITICAL:** Use validated doxycycline/tetracycline-free FBS, as traces of doxycycline/tetracycline in regular FBS may lead to undesired iPSC differentiation.

**Table:** Doxycycline Stock Solution (2 mg/mL, 1000x)

Reagent	Final concentration	Amount
Doxycycline	2 mg/mL	0.5 g
PBS, pH 7.4 (without calcium chloride, without magnesium chloride)	N/A	250 mL
**Total**	**2 mg/mL**	**250 mL**

Dissolve 0.5 g doxycycline in 25 mL PBS for a 20 mg/mL pre-dilution. Dilute required amount (e.g., 100 μL) 1:10 with PBS to 2 mg/mL and store as 50 μL aliquots at −20°C for 2–3 years. 20 mg/mL pre-dilution can be stored at −20°C and thawed at a later time point when more 2 mg/mL aliquots are needed.

**Table:** Induction Media

Reagent	Final concentration	Amount
DMEM/F-12 (Gibco 1133032)	N/A	500 mL
N2 Supplement, 100x	1x	5 mL
Non-essential Amino Acids (NEAA), 100x	1x	5 mL
GlutaMAX, 100x	1x	5 mL
**Total**	**N/A**	**515 mL**

Store at 4°C for up to 3 months. On day of use, aliquot media and add doxycycline and ROCK inhibitor fresh from stock.

**Table:** iNeuron Freezing Media

Reagent	Final concentration	Amount
BrainPhys	70%	14 mL
Knockout Serum Replacement (KOSR)	20%	4 mL
DMSO	10%	2 mL
BDNF (10 μg/mL)	10 ng/mL	20 μL
NT-3 (10 μg/mL)	10 ng/mL	20 μL
B27 supplement	1x	400 μL
**Total**	**N/A**	**20 mL**

Prepare fresh on the day of use. Adjust volumes as needed to freeze down all pre-differentiated iNeurons. Aliquots of BDNF, NT-3, and B27 can be stored at 4°C for up to two weeks.

***Alternatives:*** Normal or doxycycline/tetracycline-free FBS can be used instead of Knockout Serum Replacement (KOSR) for freezing down pre-differentiated iNeurons.

**CRITICAL:** Viability of cryopreserved iNeurons massively decreases if supplements (BDNF, NT-3, B27) are not added to freezing media.

**Table:** BDNF Stock Solution (10 μg/mL, 1000x)

Reagent	Final concentration	Amount
BDNF	10 μg/mL	10 μg
0.1% IgG- and protease-free BSA in PBS	N/A	1 mL
**Total**	**10 μg/mL**	**1 mL**

Store as 50 μL aliquots at −80°C for up to one year.

**Table:** NT-3 Stock Solution (10 μg/mL, 1000x)

Reagent	Final concentration	Amount
NT-3	10 μg/mL	10 μg
0.1% IgG- and protease-free BSA in PBS	N/A	1 mL
**Total**	**10 μg/mL**	**1 mL**

Store as 50 μL aliquots at −80°C for up to three months.

**Table:** Laminin Stock Solution (1 mg/mL, 1000x)

Reagent	Final concentration	Amount
Laminin	1 mg/mL	1 mg
PBS	N/A	lot-dependent
**Total**	**1 mg/mL**	**1 mL**

Laminin (1 mg) is shipped at lot-dependent concentrations. Add PBS to bring to a concentration of 1 mg/mL. Store as 50 μL aliquots at −80°C for up to one year.

**Table:** Uridine Stock Solution (10 mM, 1000x)

Reagent	Final concentration	Amount
Uridine	10 mM	100 mg
ddH_2_O	N/A	40.95 mL
**Total**	**10 mM**	**40.95 mL**

Filter sterilize and store at −20°C for up to one year.

**Table:** 5-Fluoro-2′-deoxyuridine Stock Solution (10 mM, 1000x)

Reagent	Final concentration	Amount
5-Fluoro-2′-deoxyuridine (FdU)	10 mM	100 mg
ddH_2_O	N/A	40.629 mL
**Total**	**10 mM**	**40.629 mL**

Filter sterilize and store at −20°C for up to one year.

**Table:** Poly-L-ornithine Stock Solution (1 mg/mL, 10x)

Reagent	Final concentration	Amount
Poly-L-ornithine	1 mg/mL	50 mg
0.1 M borate buffer	N/A	50 mL
**Total**	**1 mg/mL**	**50 mL**

Add 5 mL borate buffer to PLO bottle and swirl to ensure that all PLO is dissolved. Transfer to a 50 mL conical tube and fill up to a total volume of 50 mL. Filter sterilize and store as 1 mL aliquots at −80°C for up to one year.

**Table:** iNeuron Culture Media

Reagent	Final concentration	Amount
BrainPhys	N/A	49 mL
BDNF (10 μg/mL)	10 ng/mL	50 μL
NT-3 (10 μg/mL)	10 ng/mL	50 μL
Laminin (1 mg/mL)	1 μg/mL	50 μL
B27 supplement, 50x	1x	1 mL
**Total**	**N/A**	**50 mL**

Adjust volumes to the required amount of media and prepare fresh on the day of use. Aliquots of BDNF, NT-3, Laminin, and B27 can be stored at 4°C for up to two weeks.

**Table:** Imaging Media (per dish)

Reagent	Final concentration	Amount
Hibernate A Low-Fluorescence Medium	N/A	1.956 mL
BDNF (10 μg/mL)	10 ng/mL	2 μL
NT-3 (10 μg/mL)	10 ng/mL	2 μL
B27 supplement, 50x	1x	40 μL
**Total**	**N/A**	**2 mL**

Adjust volumes to the required amount of media and prepare fresh on the day of use. Laminin supplementation is not necessary for Imaging Media. Aliquots of BDNF, NT-3, and B27 can be stored at 4°C for up to two weeks.

### Microscopy equipment

A variety of microscopes, cameras, objectives, and acquisition software programs may be appropriate for live imaging of axonal transport. The spinning disk unit that we used can be found in the key resources table (“other” section). Listed below are the specifications of the PerkinElmer UltraView VoX spinning disk confocal system currently used in our work.^1^^,^^2^

**Table:** Microscopy system: Perkin Elmer UltraView VoX

Equipment	Equipment type
Nikon ECLIPSE TiE	Microscope stand (inverted microscope)
Nikon motorized XY stage	Stage
Nikon Ti Z drive	Focusing device (piezo)
Yokogawa CSU-X1 (50 μm pinhole)	Spinning disk unit
Hamamatsu ORCA-Fusion C14440-20UP	Camera
405, 488, 561, 640 nm	Laser lines
Excelitas X-Cite fluorescence lamp illuminator	Epifluorescence light source
ViRTEx Realtime Experiment Control Device	Photobleaching device
Nikon Perfect Focus System	Hardware-based focus maintenance device
Plan Apochromat 60x 1.40 NA	Oil immersion objective

## Step-by-step method details

### PiggyBac-mediated transfection for the stable expression of NGN2

**Timing: 1–2 weeks (for step 1)**

**Timing: 30 min (for step 2)**

**Timing: 20 min (for steps 3 and 4)**

Stable, doxycycline-inducible expression of NGN2 in a healthy population of iPSCs is a prerequisite for commencing “differentiation of iPSCs into iNeurons.” The transfection of iPSCs to accomplish this is described in this part of the protocol.

***Note:*** This protocol has been optimized for iPSCs from the KOLF2.1J parental line. Optimization of protocol conditions may be required, and subtle differences should be expected when using iPSCs from different parental lines. If using iPSCs that already stably express doxycycline-inducible NGN2 (for example WTC11 iPSCs with doxycycline-inducible NGN2 added to the safe harbor locus),^4^ proceed directly to “differentiation of iPSCs into iNeurons.”

***Note:*** Prior to starting the following steps, review and carry out the steps outlined in “preparations for iPSC culture” and “cell culture of undifferentiated iPSCs”

**Figure 1:**
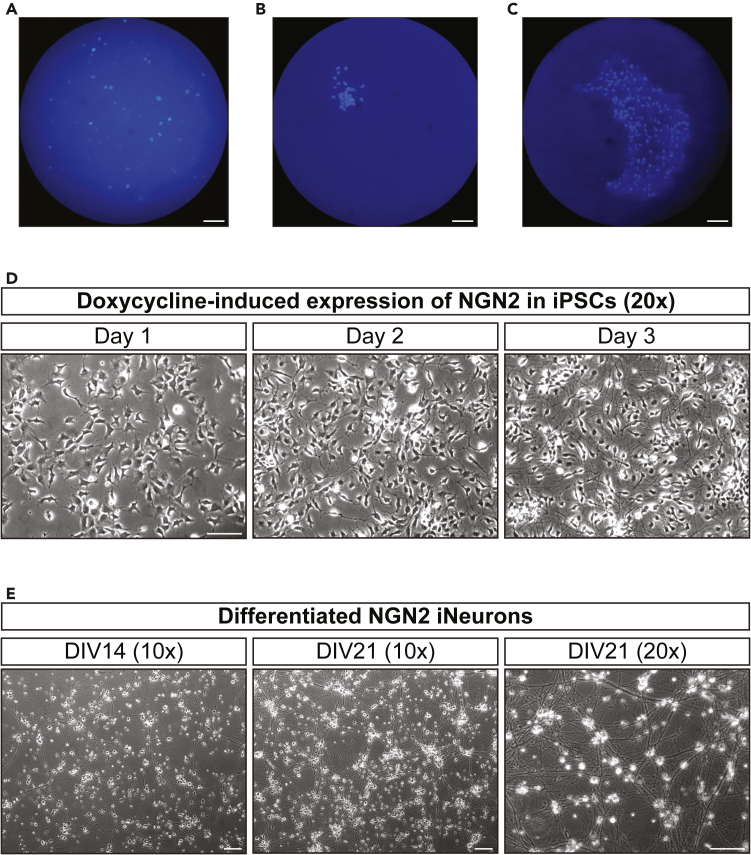
Morphology of iPSCs and differentiated iNeurons during selection and induction process (A) iPSCs imaged the morning after Lipofectamine Stem-mediated transfection with donor plasmid (PB-TO-hNGN2-puro-BFP) and EF1α-transposase plasmid. BFP+ cells should ideally be readily visible in each field of view, indicative of sufficient transfection efficiency. The image shows BFP+ cells in a field of view with confluent iPSCs. (B) iPSCs imaged 3 days after transfection. Clusters of BFP+ cells should be visible and surrounded by BFP- untransfected iPSCs, consistent with local division of healthy BFP+ cells. (C) iPSCs imaged 5 days after transfection (1 day after introducing puromycin and media change for removing heavy cell death). Isolated colonies of BFP+ cells are expected after puromycin selection. Scale bars, 100 μm. Images (A–C) were taken using a Leica DMI6000B inverted microscope. (D) iPSCs imaged at 20x magnification 1, 2, and 3 days after introduction of doxycycline into culture media. Exposure to doxycycline induces expression of hNGN2, leading to morphological changes consistent with a glutamatergic neuronal fate. Scale bar, 10 μm. (E) iNeurons imaged 14 days (left) or 21 days (middle and right) after plating at 10x or 20x magnification. Scale bar, 10 μm. Images (D–E) were taken using a Zeiss Axio Observer Z1 microscope with Plan-Apochromat 10x/0.45NA and LD Plan-Neofluar 20x/0.4NA objectives.

1. Following ReLeSR passaging (see “cell culture of undifferentiated iPSCs”), feed iPSCs daily with 10 mL Essential 8.
  ***Note:*** Do not add RI after ReLeSR passaging.
  a. Visually assess cultures daily using a tissue culture microscope to monitor growth until again at 70–80% confluency.
    ***Note:*** Cells can continue to be passaged before starting Piggybac-mediated transfection in step 3; a minimum of one passaging after thawing is required.
2. Split iPSCs using Accutase and plate iPSCs for Piggybac-mediated transfection.
  a. Aliquot 6 mL of Accutase reagent and place in 37°C water bath.
  b. Aspirate Essential 8 media and rinse dish twice with 5 mL PBS (without Ca^2+^ and Mg^2+^).
  c. Add 3 mL pre-warmed Accutase to the dish and place in 37°C incubator for 10 min.
  d. Tilt the dish and pipette the Accutase solution 5x down the culture surface with a 5 mL serological pipette.
  e. Transfer cells from the dish to a 15 mL conical tube and pipette up and down 10-15x to break apart clumps.
  f. Rinse culture surface with 2 mL Essential 8 and combine with the cell suspension.
  g. Pipette up and down 5-10x with a 5 mL serological pipette to further break apart cell clumps.
  h. Centrifuge tube at 200 g for 4 min.
  i. Aspirate supernatant and resuspend pellet in 1 mL Essential 8 + RI by gently pipetting up and down 5-10x with a P1000 pipette.
  j. Add Essential 8 + RI to a total volume of 5 mL and mix by pipetting up and down with a 5 mL serological pipette.
  k. Count live cells using Trypan blue. Suspend 800k iPSCs in 2 mL Essential 8 + RI.
  l. Aspirate Matrigel solution from the 6-well dish and immediately add the 2 mL cell suspension to one well.
  m. Place dish in 37°C incubator and move front-to-back and side-to-side several times to evenly distribute cells.
3. Perform Piggybac-mediated transfection for doxycycline-inducible expression of NGN2.
  a. 3–6 h after iPSC plating, confirm that cells appear healthy and attached using a tissue culture microscope.
  b. Prepare two 1.5 mL Eppendorf tubes:
    Tube A: add 0.75 μg donor plasmid (PB-TO-hNGN2-puro-BFP) and 0.37 μg EF1α-transposase plasmid to 100 μL OptiMEM media.
    Tube B: add 4 μL Lipofectamine Stem to 100 μL OptiMEM media.
    ***Note:*** A 2:1 ratio of donor:transposase is preferred. The above transfection conditions work best in our hands, but transfection conditions may require optimization, and achieving a good transfection efficiency is important for subsequent selection steps. Consider attempting transfections with different DNA amounts (maintaining 2:1 donor:transposase ratio) in multiple wells in parallel and keeping the well with the highest transfection efficiency (Troubleshooting 1 and Troubleshooting 2).
  c. Transfer OptiMEM + Lipofectamine Stem from Tube B to Tube A and mix well by pipetting up and down 5x with a P200 pipette.
  d. Incubate Lipofectamine-DNA mix for 10 min at RT (18°C–22°C).
  e. Add Lipofectamine-DNA mix dropwise to well with iPSCs.
  f. Gently swirl plate to mix and return to incubator.
4. Culture Piggybac-transfected iPSCs stably expressing doxycycline-inducible NGN2.
  a. The morning after transfection, use a fluorescence microscope to check for transfection efficiency.
    ***Note:*** The well should be quite dense with healthy iPSCs and minimal cell death. Transfection efficiency can be estimated based on the % of blue cells (Figure 1A). If transfection efficiency is very low or cell death is high, consider repeating the preceding steps (Troubleshooting 1 and Troubleshooting 2).
  b. Use Accutase to passage all cells from transfected well to a 10 cm Matrigel-coated dish, in Essential 8 + RI.
  c. The next day, replace Essential 8 + RI with fresh Essential 8 without RI.
  d. Continue to culture iPSCs with daily media changes. Each day, use a fluorescence microscope to evaluate BFP+ cells.
    ***Note:*** Local division of BFP+ cells should be observed, forming small clusters (Figure 1B). At this stage, many untransfected BFP- cells will be present, surrounding the subset of BFP+ cells.
  e. When iPSCs (including both BFP+ and BFP- cells) are ∼40–60% confluent, introduce puromycin to Essential 8 at a dose of 0.5 μg/mL.
    ***Note:*** This dose of puromycin works best in our hands for KOLF2.1J iPSCs. Appropriate puromycin dosing may vary dramatically for iPSCs from different parental lines. Optimization of puromycin dose is recommended if results of selection are not satisfactory. We recommend using 1.5x the lowest dose that kills 100% of untransfected wild-type iPSCs overnight (12–16 hours) (Troubleshooting 3).
  f. Check cells after 12–16 h using a tissue culture microscope.
    ***Note:*** Heavy cell death is expected as a result of puromycin selection.
  g. Change Essential 8 + puromycin.
  h. Using a tissue culture microscope, check cells again after media change.
    ***Note:*** Confirm that individual colonies of surviving, morphologically healthy iPSCs remain. Use a fluorescence microscope to confirm that remaining colonies are BFP+ (Figure 1C). To evaluate the purity of the selected colonies, switch back and forth between bright field and fluorescence and compare the boundaries of the colony.
  i. The following day, once purity of colonies is visually confirmed, change to Essential 8 media without puromycin.
  j. Continue daily media changes and expand iPSCs to ∼70% confluency.
  k. Freeze down iPSCs and store in liquid nitrogen.
    ***Note:*** This preserved stock of iPSCs will be stably expressing NGN2, puromycin-resistance, and BFP. Given the labor-intensive nature of performing the Piggybac transfection and selection, we recommend freezing a large stock of low passage number iPSCs at this stage. Many iNeuron inductions can be performed from the same batch of Piggybac transfected iPSCs.
    i. Prepare iPSC Freezing Media and label cryovials.
    ii. Aliquot 5 mL Accutase and place in water bath to warm up to 37°C.
    iii. Aspirate Essential 8 media and rinse cells twice with 5 mL PBS (without Ca^2+^ and Mg^2+^).
    iv. Add 5 mL Accutase and place in 37°C incubator for 5 min.
    v. Tilt the 10 cm dish and pipette the Accutase solution 5x down the culture surface with a 5 mL serological pipette.
    vi. Transfer cells to a 15 mL conical tube and pipette up and down gently to break apart clumps.
    vii. Rinse culture surface with 5 mL Essential 8 media + RI and combine with the cell suspension.
    viii. Centrifuge tube at 1000 g for 5 min.
    ix. Aspirate supernatant and resuspend pellet in 1 mL iPSC Freezing Media by pipetting up and down gently 5-10x with a P1000 pipette.
    x. Add iPSC Freezing Media to a total volume of 5 mL and mix by pipetting up and down with a 5 mL serological pipette.
    xi. Count cells. Dilute cells to a final concentration of 1 million cells per mL using iPSC Freezing Media.
    xii. Transfer cells into cryotubes (1 mL aliquots).
      ***Note:*** Work quickly to avoid prolonged exposure to DMSO.
    xiii. Freeze down cells in a Mr. Frosty container placed in a −80°C freezer overnight (12–16 h).
  l. On the following day, transfer cryopreserved iPSCs to liquid nitrogen for long-term storage.
    **Pause point:** iPSCs stably expressing doxycycline-inducible NGN2 can be cryopreserved in liquid nitrogen.

### Differentiation of iPSCs into iNeurons

**Timing: 8–10 days (for steps 5 and 6)**

**Timing: 1 h (for step 7)**

**Timing: 30 min (for step 8)**

**Timing: 30 min (for step 9)**

**Timing: 2–3 h (for step 10)**

This part of the protocol describes the differentiation of iPSCs stably expressing doxycycline-inducible NGN2 upon Piggybac-mediated delivery into iNeurons. The steps described below have been modified from a previously published protocol for the differentiation of iPSCs from the WTC11 parental line expressing doxycycline-inducible NGN2 in the AAVS1 safe harbor locus.^4^

***Note:*** This protocol has been optimized for iPSCs from the KOLF2.1J parental line upon Piggybac-mediated delivery of doxycycline-inducible NGN2. Optimization of protocol conditions may be required, and subtle differences should be expected when using iPSCs from different parental lines.

***Note:*** Prior to starting the following steps, review and carry out the steps outlined in “preparations for iPSC culture” and “preparations for differentiation of iPSCs into iNeurons.”

5. Thaw and culture iPSCs stably expressing doxycycline-inducible NGN2 (see “cell culture of undifferentiated iPSCs”).
6. Following ReLeSR passaging, feed iPSCs daily with 10 mL Essential 8.
  ***Note:*** Do not add RI after ReLeSR passaging.
  a. Visually assess cultures daily using a tissue culture microscope to monitor growth until again at 70–80% confluency.
    ***Note:*** Cells can continue to be passaged before starting differentiation into iNeurons; a minimum of one passaging after thawing is required**.**
7. Day 0 of iNeuron induction: split iPSCs using Accutase and plate in Induction Media containing doxycycline and RI.
  ***Note:*** The steps below describe the differentiation of one 15 cm dish of iNeurons. This can be scaled up if a larger number of iNeurons is required.
  a. Add 30 μL Doxycycline Stock Solution and 30 μL ROCK inhibitor Stock Solution to 30 mL Induction Media and place in 37°C water bath.
    ***Note:*** Final concentration of doxycycline in Induction Media is 2 μg/mL. Final concentration of ROCK inhibitor in Induction Media is 10 μM.
  b. Aliquot 6 mL of Accutase reagent and place in 37°C water bath.
  c. Aspirate Essential 8 media and rinse each dish twice with 5 mL PBS (without Ca^2+^ and Mg^2+^).
  d. Add 3 mL pre-warmed Accutase to each dish and place in 37°C incubator for 10 min.
  e. Tilt the dishes and pipette the Accutase solution about 5x down the culture surface with a 5 mL serological pipette.
  f. Transfer cells from both dishes to a 15 mL conical tube and pipette up and down 10-15x to break apart clumps.
  g. Rinse culture surface with 2 mL Induction Media (+ doxycycline, + RI) per dish and combine with the cell suspension.
  h. Pipette up and down 5-10x with a 5 mL serological pipette to further break apart cell clumps.
  i. Centrifuge tube at 200 g for 4 min.
  j. Aspirate supernatant and resuspend pellet in 1 mL Induction Media (+ doxycycline, + RI) by gently pipetting up and down 5-10x with a P1000 pipette.
  k. Add Induction Media (+ doxycycline, + RI) to a total volume of 5 mL and mix by pipetting up and down with a 5 mL serological pipette.
  l. Count cells and transfer 4.5 million cells to a 50 mL tube.
  m. Add Induction Media (+ doxycycline, + RI) to a total volume of 20 mL and mix by pipetting up and down with a 25 mL serological pipette.
  n. Aspirate Matrigel from the 15 cm dish and immediately add the 20 mL cell suspension.
  o. Place 15 cm dish in 37°C incubator and move front-to-back and side-to-side several times to evenly distribute cells.
8. Day 1 of iNeuron induction: feed cells with Induction Media containing doxycycline.
  a. Check cells under the microscope to assess morphology.
    ***Note:*** Cells should show spike-like extrusions resulting from RI treatment and nascent neuritic extensions (Figure 1D).
  b. Prepare 20 mL Induction Media (+ doxycycline, without RI) and place in water bath for approximately 20 min to warm up to 37°C.
  c. Aspirate old Induction Media from 15 cm dish and replace with pre-warmed fresh Induction Media (+ doxycycline, without RI).
9. Day 2 of iNeuron induction: feed cells with Induction Media containing doxycycline.
  a. Check cells under the microscope to assess morphology.
    ***Note:*** Neurite extensions should be evident at this point (Figure 1D).
  b. Prepare 20 mL Induction Media (+ doxycycline, without RI) and place in water bath for approx. 20 min to warm up to 37°C.
  c. Aspirate Induction Media from 15 cm dish and replace with pre-warmed Induction Media (+ doxycycline).
10. Day 3 of iNeuron induction: harvest pre-differentiated iNeurons and freeze down for liquid nitrogen storage.
  a. Check cells under the microscope to assess morphology.
    ***Note:*** Neurite extensions should be clearly visible (Figure 1D). If neuritic extensions are not present or cells appear unhealthy, cells should be discarded, and the protocol should be re-started with a new batch of iPSCs and new Induction Media with fresh doxycycline and RI (Troubleshooting 4).
  b. Prepare iNeuron Freezing Media and label cryovials. Aliquot 10 mL Accutase and place in water bath to warm up to 37°C.
    ***Note:*** The required volume of Freezing Media depends on the concentration at which iNeurons are to be frozen down and on the yield of pre-differentiated iNeurons. For live imaging experiments using MatTek 35 mm imaging dishes, we recommend freezing down 1 mL aliquots at a concentration of 1 million cells per mL. Typically, one 15 cm dish yields about 15 million pre-differentiated iNeurons.
  c. Aspirate Induction Media and rinse cells twice with 10 mL PBS (without Ca^2+^ and Mg^2+^).
  d. Add 10 mL Accutase and place in 37°C incubator for 10 min.
  e. Tilt the 15 cm dish and pipette the Accutase solution 5x down the culture surface with a 10 mL serological pipette.
  f. Transfer cells to a 15 mL conical tube and pipette up and down 10-15x to break apart clumps (Troubleshooting 5).
  g. Rinse culture surface with 3 mL BrainPhys and combine with the cell suspension.
  h. Centrifuge tube at 1000 g for 5 min.
  i. Aspirate supernatant and resuspend pellet in 1 mL iNeuron Freezing Media by pipetting up and down gently 5-10x with a P1000 pipette.
  j. Add iNeuron Freezing Media to a total volume of 7 mL and mix by pipetting up and down with a 5 mL serological pipette.
  k. Count cells. Dilute cells to a final concentration of 1 million cells per mL using iNeuron Freezing Media.
  l. Transfer cells into cryotubes (1 mL aliquots).
    ***Note:*** Work quickly to avoid prolonged exposure to DMSO.
  m. Freeze down cells in a Mr. Frosty container placed in a −80°C freezer overnight (12–16 h).
  n. On the following day, transfer cryopreserved neurons to liquid nitrogen for long-term storage.
    **Pause point:** iNeurons can be cryopreserved in liquid nitrogen. In our hands, iNeuron viability decreases after approximately one year of liquid nitrogen storage.

### Cell culture of iNeurons

**Timing: 2–3 weeks**

**Timing: 45 min (for step 12)**

**Timing: 30 min (for step 13)**

**Timing: 1 h (for step 14)**

**Timing: 1 h (for step 15)**

This part of the protocol describes the maintenance and culture of iNeurons after differentiation toward a glutamatergic neuronal fate has been completed (see “differentiation of iPSCs into iNeurons”).

***Note:*** Prior to starting the following steps, review and carry out the steps outlined in “preparations for iNeuron culture”.

11. On the day of plating (day *in vitro* 0, DIV0), prepare 3 mL iNeuron Culture Media containing 10 μM 5-Fluoro-2′-deoxyuridine (FdU) and 10 μM uridine per each imaging dish.

***Note:*** It is not necessary to warm the media, as it will be warmed on the imaging dishes.

***Note:*** For iPSCs that have NGN2 expression via the Piggybac method (“PiggyBac-mediated transfection for the stable expression of NGN2”), a small number of cells have been observed to remain mitotically active following neuronal induction. These mitotically active cells are barely detectable at earlier DIVs but are easily identified as cell colonies at later time points due to their continued growth over the course of weeks in long-term neuronal cultures. To prevent growth of mitotically active cells, inclusion of anti-mitotic drugs in the culture media is advised. In our hands, an overnight (12–16 hour) pulse at the time of plating with 10 μM FdU and 10 μM uridine, followed by full media replacement, was highly effective in preventing growth of mitotic cells. Inclusion of FdU and uridine in media at time of plating is not necessary for iPSCs with NGN2 in the AAVS1 safe harbor locus.

12. Move PLO-coated imaging dishes from 37°C incubator to tissue culture hood for washes with ddH_2_O and addition of iNeuron Culture Media.
  a. Aspirate 1x PLO solution from imaging dishes.
  b. Add 1.5 mL of ddH_2_O to each dish.
    ***Note:*** This will comfortably cover the PLO-coated glass center of each dish.
  c. Aspirate ddH_2_O from each dish. Repeat for a total of two washes with ddH_2_O.
  d. Aspirate ddH_2_O from each dish, carefully removing excess droplets.
  e. Allow dishes to dry in tissue culture hood for several minutes.
  f. Add 2 mL of iNeuron Culture Media containing 10 μM FdU and 10 μM uridine to each imaging dish.
  g. Move dishes to culture incubator for a minimum of 30 min.
    ***Note:*** This time period allows for warming and CO_2_ equilibration of the media.
13. Thaw pre-differentiated iNeurons and plate on imaging dishes.
  a. Transport vials of iNeurons on ice from liquid nitrogen storage.
  b. For best viability, quickly thaw vials in a 37°C water bath.
  c. Move vial to tissue culture hood when only a small amount of ice remains visible.
  d. Transfer contents of vial (∼1 mL) to 15 mL conical tube.
  e. Add 1 mL of iNeuron media containing 10 μM FdU and 10 μM uridine.
  f. Centrifuge at 1000 g for 4 min. Aspirate supernatant in the tissue culture hood.
  g. Using p1000 pipettor, gently resuspend cell pellet with 750 μL of RT (18°C–22°C ) iNeuron media containing 10 μM FdU and 10 μM uridine.
    ***Note:*** When resuspending, use minimal force to dissolve chunks until solution appears homogeneous (Troubleshooting 5).
  h. Perform live cell count using Trypan blue.
  i. For live axonal imaging, plate 300,000 iNeurons per dish.
    i. Add iNeurons dropwise over glass center of imaging dish.
      ***Note:*** Do not shake or mix the dish, as the neurons will only grow on the PLO coated center of the dish in the presence of laminin-supplemented media.
    ii. Return dishes to culture incubator.
14. The day after plating (DIV1), replace the full volume of the culture media with iNeuron Culture Media without FdU and uridine.
  a. Prepare 2 mL iNeuron Culture Media per dish without anti-mitotic drugs.
  b. Warm media in a sterile dish or flask in culture incubator for a minimum of 30 min.
    ***Note:*** If using a flask, be sure to keep lid ajar (gently resting on the flask opening) in order to allow for incubator gas exchange with the media.
  c. Gently aspirate media containing anti-mitotic drugs from imaging dishes.
  d. Remove excess media but do not fully dry the dish.
    ***Note:*** Take care to not directly touch the glass center of the dish in order to avoid disrupting neuronal attachment.
  e. Replace media with 2 mL fresh, warmed iNeuron Culture Media.
15. Every 3–4 days, perform partial media change.
  a. In the incubator, warm 800 μL complete iNeuron Culture Media per dish for 30 min in a tissue culture dish or culture flask.
    ***Note:*** This includes a small volume of extra media to account for evaporation in the incubator. If using a flask, be sure to keep lid ajar (gently resting on the flask opening) in order to allow for incubator gas exchange with the media.
  b. Move imaging dishes with developing iNeurons to tissue culture hood.
  c. Using a p1000 pipettor, gently remove 600 μL from each dish, aspirating only from the edge of the dish.
  d. Add 750 μL of fresh, pre-equilibrated iNeuron media.
16. Allow iNeurons to develop until DIV14-21 (Figure 1E), or later depending on experimental needs.

***Note:*** We performed live imaging experiments at DIV21, and fixed immunofluorescence imaging at either DIV14 or DIV21. However, optimization and characterization of protein expression at different DIV may be required for specific experiments. In our hands, maintaining attachment of iNeurons to PLO coated dishes becomes more challenging at time points exceeding DIV21 (Troubleshooting 6).

### Transfection of iNeurons for the expression of fluorescently labeled axonal cargoes

**Timing: 3 h**

This part of the protocol describes how cultured iNeurons are transfected using Lipofectamine Stem transfection reagent. For live imaging experiments, iNeurons are typically transfected with plasmids encoding fluorescently labeled proteins approximately 48–72 h prior to imaging on DIV21.

17. For each imaging dish, prepare 2.5 mL fresh iNeuron Culture Media.
18. Transfer fresh iNeuron media to a 10 cm dish and place in 37°C incubator to equilibrate for at least 20 min.
19. Prepare two 1.5 mL Eppendorf tubes for each imaging dish:

Tube A: add 1 μg plasmid DNA to 100 μL OptiMEM media.

Tube B: add 4 μL Lipofectamine Stem to 100 μL OptiMEM media.

***Note:*** Transfection conditions may require optimization depending on the specific plasmid(s) used in an experiment (Troubleshooting 7). A typical transfection with one plasmid uses 1 μg of plasmid DNA and 4 μL Lipofectamine Stem. For co-transfection of two plasmids, use 2 μg of plasmid DNA (1 μg each plasmid) and 5 μL Lipofectamine Stem as a starting point for further optimization.

**CRITICAL:** Plasmids with PGK or EF1alpha promoters express best in iNeurons. In our hands, plasmids with CMV promoter do not express reliably in iNeurons.

20. Transfer OptiMEM + Lipofectamine Stem from Tube B in Tube A and mix well by pipetting up and down 5x with a P200 pipette.
21. Incubate Lipofectamine-DNA mix for 10 min at RT (18°C–22°C).
22. Remove conditioned media from imaging dishes and immediately replace with fresh pre-equilibrated iNeuron Culture Media.
23. Save conditioned media in 37°C incubator for later.
24. Add 500 μL fresh iNeuron Culture Media to every 2 mL of conditioned media.
25. Add Lipofectamine-DNA mix dropwise to each imaging dish.
26. Place imaging dishes in 37°C incubator for 90 min.
27. Aspirate Lipofectamine-DNA mix containing media and replace with the conditioned media collected earlier.
28. Return imaging dishes to 37°C incubator.
29. Image 48–72 h after transfection.

### Live imaging of iNeurons for the recording of axonal transport

**Timing: 1–8 h**

This part of the protocol describes how live imaging with confocal microscopy can be performed to generate recordings of axonal transport of fluorescently-labeled cargoes in iNeurons.

A variety of microscopes, cameras, objectives, and acquisition software programs may be appropriate for live imaging of axonal transport. Listed in the “materials and equipment” section are the specifications of the spinning disk confocal system currently used in our work.^1^^,^^2^

***Note:*** Prior to starting the following steps, review and carry out the steps outlined in “transfection of iNeurons for the expression of fluorescently labeled axonal cargoes”.

30. On the day of imaging (iNeurons DIV21, 48–72 h after transfection), prepare 2 mL Imaging Media for each imaging dish and warm by placing in a 37°C waterbath.

***Note:*** Laminin supplementation is not necessary for Imaging Media.

31. Warm environmental chamber of the confocal microscope to 37°C.

***Note:*** It is important to maintain steady temperature during the imaging session as well as across different replicates.

32. Remove one imaging dish with transfected DIV21 iNeurons from the culture incubator.
33. Tilt the dish and, using a p1000 pipette, gently aspirate and discard the culture media, leaving only a small volume.
34. Gently replace the media with warmed Imaging Media (prepared in step 30).

***Note:*** Take care to not detach sheet of neurons with forceful pipetting.

35. Immediately begin imaging with spinning disk confocal microscope.

***Note:*** Limit imaging of each imaging dish to roughly 1 hour. Despite buffering from imaging media and temperature control from the environmental chamber, neuronal health may suffer after longer periods outside the incubator.

***Note:*** For live imaging of axonal cargoes, a 60x oil-immersion objective is recommended. This provides sufficient resolution while also allowing for a field of view that can accommodate a long stretch of axon.

***Note:*** Activating a focus drift prevention system (such as the Nikon Perfect Focus System) is strongly recommended. The thin caliber of axons makes it challenging to keep stretches of axon in frame if any drift is present during time lapse imaging.

***Note:*** Assess the brightness of fluorescently-labeled cargoes and adjust imaging parameters accordingly (see also step 37, “cargo-specific parameters.”) It is preferred to keep laser power and framerate constant across different experimental replicates to limit variability, but some optimization is necessary during the first replicate or pilot experiment. Aim for the lowest laser power that provides high signal to noise ratio in order to minimize photobleaching.

36. Follow guiding principles for axonal imaging (see Notes).

***Note:*** Identification of axons - Given the key differences in transport dynamics for organelles in axons compared to dendrites, it is essential to accurately distinguish between axons and dendrites. This can be challenging for an inexperienced investigator due to the heterogeneity in the appearance of cultured neurons. At DIV21, the axon in healthy WT iNeurons can be identified with high fidelity as the longest neuronal process. In addition, the axon typically maintains uniform width, where dendrites tend to taper from soma to end. It is recommended to take the time to assess multiple processes of a neuron by following them to their termini in situations of uncertainty. It may be beneficial to first become familiar with the morphology of fixed DIV21 iNeurons with immunostaining of axonal (e.g. Ankyrin G) and dendritic (e.g. MAP2) markers.

***Note:*** Identification of directionality - The direction of cargo movement in the axon relative to the soma and the axon terminal has important biological relevance and is crucial to document. We strongly recommend confirming the orientation of the axon before starting recording. This can be accomplished by starting at a known neuronal soma and following the axon outward to choose a field of view (see step 36c, “Selecting a field of view” below). Great care must be taken when tracing axons, as there may be ambiguity at intersections with other fluorescently-labeled processes. Strategies for resolving uncertainty when tracing an axon through intersections include careful observation while changing the Z-axis, waiting for a fluorescent cargo to pass through, and looking for apparent differences in fluorescence intensity between different transfected neurons. If it is not possible to reliably trace an axon, it should be abandoned, and a new axon should be selected. Once the orientation has been established, this should be documented on a simplified drawing of the field of view.

***Note:*** Selecting a field of view - Choice of the field of view prior to time lapse recording affects the ability to generate high quality kymographs that are easy to interpret for analysis of trafficking parameters. In particular, it is important to find an axon segment that remains in the same focal plane for a long, uninterrupted stretch. A good rule of thumb is to take the time to look for an axon segment that is continuously in focus for >50% of the total field of view at 60x magnification. As mentioned above, employing a focus drift prevention system is essential to keep the axon segment in focus during time-lapse acquisition.

37. Consider cargo-specific parameters and tips (see Notes).

***Note:*** Autophagosomes - We image autophagosome transport in iNeuron axons by expressing LC3B labeled with either EGFP (Addgene #200427) or mScarlet (Addgene #200083).^1^ LC3B is not only conjugated to the autophagosome membrane but also present in the cytosol, resulting in a cytosolic “background” signal in neurons overexpressing fluorescently-labeled LC3B. Axons with overly bright cytoplasmic signal should be avoided, as this makes it challenging to clearly track the motility of LC3B+ vesicles. Axons should be evaluated for clearly visible punctate LC3B signal prior to beginning time-lapse recording. For autophagosomal transport, we typically image using a 60x objective and record at a framerate of 1 frame per second for 5 min.

***Note:*** Mitochondria - We visualize mitochondria by expressing the mitochondrial targeting sequence of COX8 labeled with fluorescent proteins (e.g. Mito-mEmerald; Addgene #200430). Fluorescently labeled mito constructs typically result in a very low cytoplasmic background signal. The correct path of the axon can be determined by temporarily increasing the gain (to brighten the faint cytoplasmic signal) or co-transfecting iNeurons with a cytoplasmic filler with a different fluorescence wavelength. Alternatively, it may be helpful to wait for motile mitochondria to pass through ambiguous intersections to elucidate the correct path of the axon. For mitochondrial transport, we typically image using a 60x objective and record at a framerate of 1 frame per second for 5 min.

***Note:*** Synaptic vesicle precursors - We visualize synaptic vesicle precursors (SVPs) by expressing synaptophysin (Syp) labeled with fluorescent mScarlet (Addgene #206145). Our group has also visualized SVPs using fluorescently-labeled synaptobrevin-2 with equivalent success.^7^ Compared to cargoes previously discussed, the anterogradely moving population of Syp+ vesicles is small in diameter and rapidly moving. To maximize visibility of this population, we recommend using mScarlet or an equivalently bright and rapidly-maturing protein. Finally, cytoplasmic synaptophysin signal in the axon may be bright, reducing signal to noise ratio for tracking SVP transport. If this is the case, we recommend briefly photobleaching the selected stretch of axon immediately prior to imaging, using a ViRTEx Realtime Experiment Control Device or equivalent tool. In our hands, the axonal mScarlet-Syp signal was readily photobleached with brief (3 ms/pixel) exposure with the 405 nm laser. While the background signal slowly recovered from bleaching over the following minutes, this was sufficient to generate high-fidelity kymographs depicting unambiguous SVP transport. For SVP transport, we typically image using 60x magnification and record at a relatively fast framerate of at least 4 frames per second for a total of 5 min (including photobleaching time). At lower framerates, a fraction of small, rapid vesicles will not be detected, impairing proper characterization of velocity and processivity in the population.

## Expected outcomes

This protocol describes differentiation, plating, culture, and transfection of iPSC-derived glutamatergic iNeurons for live imaging of axonal transport. These techniques have been used for robust data collection to characterize the transport of autophagosomes, mitochondria, and synaptic vesicle precursors.^1^^,^^2^ Analysis of various transport parameters is illustrated in detail in the two cited primary research manuscripts.^1^^,^^2^ Cargo transport parameters that can be observed and analyzed with high resolution and fidelity include, but are not limited to: size, flux, directionality, velocity, pause number, pause duration, reversal number, and run length. In addition to live imaging experiments of axonal transport, iNeurons can be fixed and permeabilized for immunofluorescence studies.^2^ iNeurons can likely also be used for a wide range of assays other than specified here, which may however require optimization of culture length and plating conditions.

## Quantification and statistical analysis

**Figure 2:**
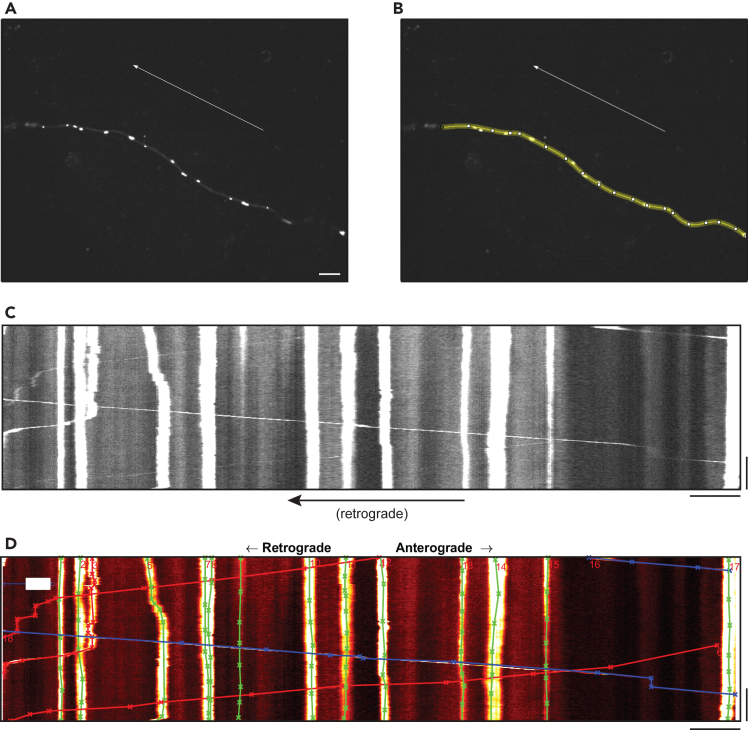
Kymograph plotting and tracing for motility analysis (A) Still frame from axonal time-lapse imaging of a DIV21 WT iNeuron expressing mito-mEmerald for visualization of mitochondria. Arrow (added by investigator in ImageJ) indicates retrograde direction (toward soma). Scale bar, 10 μm. (B) Example region of interest (ROI) traced over axon in ImageJ by investigator. This ROI is subsequently used by the Multi Kymograph functionality in ImageJ to generate a kymograph (C), with distance along the axonal ROI plotted on the X-axis and time plotted on the Y-axis. Scale bars, 10 μm (x-axis) and 60 s (y-axis). (D) Manual tracing of mitochondrial motion performed by investigator using custom MATLAB software (KymoSuite). The raw time-lapse video should be reviewed while performing tracing to resolve ambiguities in the kymograph (e.g., one vs. two mitochondria at the same location at a given time point). Scale bars, 10 μm (x-axis) and 60 s (y-axis).

Axonal transport data collected in the form of time-lapse recordings can be quantified in different ways to best suit the experiment in question. We recommend generating kymographs from the raw videos in order to best visualize transport and aid in quantification. For our workflow, we first use the “Multi Kymograph” functionality of ImageJ to generate kymographs for a region of interest along an axon, taking care to keep anterograde-retrograde directionality consistent (Figures 2A–2C). Multiple tools exist for manual or semi-automated tracing of kymographs.^8^ Our group most frequently uses custom MATLAB software (KymoSuite^9^; see key resources table) for manual tracing of kymographs, giving the investigator the most agency to ensure tracings that best represent the raw time-lapse data (Figure 2D). The raw time-lapse video should be reviewed while performing tracing to resolve ambiguities in the kymograph (e.g., one vs. two mitochondria at the same location at a given time point). Semi-automated tracing tools can be considered especially if the number of tracings is prohibitively high to be done manually.

## Limitations

Particularly for live imaging experiments, differentiation of iPSCs into iNeurons by induced expression of the transcription factor NGN2 offers several advantages. Within a relatively short time, this approach consistently yields morphologically mature glutamatergic neurons that show very similar characteristics of axonal and dendritic organelle transport as compared to primary hippocampal neurons.^10^ An inherent limitation is that translation of this differentiation approach to other neuronal types requires careful protocol development and characterization. Thus far, an analogous differentiation protocol has already been developed for motor neurons and is in use by multiple groups.^6^^,^^11^ Ongoing work in the field continues to yield NGN2-based differentiation protocols for other neuronal subtypes such as dopaminergic neurons.^12^ Due to differences in plating and culture, our live imaging protocol is optimized specifically to glutamatergic iNeurons.

The Lipofectamine-mediated transfection of iNeurons described here typically results in a relatively low percentage of cells expressing the transfected plasmid. This is well suited for live imaging experiments, as fluorophore expression in a small number of iNeurons makes it easy to trace individual neurites and distinguish dendrites and axons by length. If a higher percentage of neurons expressing the plasmid of interest is required, other transfection methods or viral-mediated delivery may need to be used. In addition, we found that co-expression of >2 plasmids becomes more challenging to achieve by Lipofectamine Stem transfection and may instead require other methods of plasmid delivery.

## Troubleshooting

### Problem 1

Low transfection efficiency during Piggybac-mediated transfection.

### Potential solution

• While a wide range of transfection efficiencies can be sufficient, we recommend optimizing this step if BFP-expressing cells are rare (only few per field of view). A low transfection efficiency results in very sparse colonies after the puromycin selection step, which may be more challenging to keep healthy during a long expansion process.
• In our hands, we achieved best results with transfection conditions of 0.75 μg donor (PB-TO-hNGN2-puro-BFP), 0.37 μg EF1α-transposase, and 4 μL Lipofectamine Stem (see Figure 1A for example transfection efficiency). If the initial attempt has markedly lower transfection efficiency, consider attempting transfections in multiple parallel wells with adjusted DNA amounts, while maintaining the 2:1 donor:transposase ratio. These wells can be evaluated the morning following the transfection, with the well with the highest transfection efficiency being kept for subsequent steps (step 4).
• If transfection efficiency appears to be low but rare, very brightly blue iPSCs are observed, overly high expression could have resulted in die-off of transfected cells. Consider reducing quantity of transfected DNA (step 3).

### Problem 2

High amount of cell death during Piggybac-mediated transfection.

### Potential solution

A small amount of cell death is expected following Lipofectamine Stem-mediated transfection of iPSCs, but cell density in the well should be high the morning following transfection. If cell death is excessive (regardless of transfection efficiency), most likely the ratio of transfection reagent to number of plated cells is too high. Ensure that starting cell count is correct and that starting iPSCs are healthy; if so, consider reducing quantity of Lipofectamine Stem, but be cautious as this may also reduce transfection efficiency (step 3).

### Problem 3

Failure to select for healthy blue cells using puromycin after Piggybac transfection.

### Potential solution

In our hands, puromycin selection may be less effective if iPSCs are too dense. We recommend attempting puromycin selection when cells are a maximum of ∼40–60% confluent.

The recommended dose of puromycin (0.5 μg/mL) is specifically for KOLF2.1J iPSCs. Appropriate puromycin dosing may vary dramatically for iPSCs from different parental lines. Optimization of puromycin dose is recommended if results of selection are not satisfactory. The proper dose can be ascertained by treating wild-type, untransfected iPSCs with ascending doses of puromycin in parallel wells of a 6-well plate, using one well without puromycin as a negative control. We recommend using 1.5x the lowest dose that kills 100% of untransfected wild-type iPSCs overnight (12–16 h), and longer periods of selection with puromycin may also be used.

### Problem 4

High amount of cell death during iNeuron differentiation.

### Potential solution

• If there is doubt about the health or quality of differentiation, it is strongly recommended to repeat differentiation process rather than proceed with the dissociation and freezing steps. Discard cells and repeat with a new batch of iPSCs and freshly made Induction Media.
• Increase the number of iPSCs plated on a 15 cm dish for iNeuron differentiation (step 7). Plating of too few cells for differentiation results in high cell death and incomplete differentiation.

### Problem 5

Difficulty with dissociating differentiated neurons from 15 cm dish, or overly clumped iNeurons at time of plating.

### Potential solution

• Decrease the number of iPSCs plated on a 15 cm dish for iNeuron differentiation (step 7).
• Increase incubation time with Accutase (step 10).
• Ensure that Accutase is warm for best dissociation results (step 10). This can be optimized in several ways. Allow adequate time for Accutase to warm in the water bath before starting dissociation. If sharing tissue culture room time with colleagues, be strategic about which incubator to perform Accutase incubation in, as repeated door openings by colleagues during the incubation time may reduce dissociation efficacy. Also, if incubating multiple dishes at once, place dishes in a single layer with each getting direct contact with metal incubator shelf. In our hands, dishes stacked on top of each other have more variable dissociation outcomes.

### Problem 6

iNeurons detach from live imaging dishes.

### Potential solution

• Use high quality glass bottom live imaging dishes. We found that iNeurons adhere better to MatTek glass bottom dishes compared to those of other companies.
• Make sure that PLO coating is performed at 37°C and overnight (12–16 h) (preparation 13).
• Let PLO-coated dishes dry for several minutes after washing with ddH_2_O (step 12).
• Adding Matrigel at 1:200 dilution in one of the weekly feeds helps to keep iNeurons attached for longer culture durations (step 15).
• Consider performing experiments at earlier DIV, depending on the cargoes/proteins of interest for the specific experiment.

### Problem 7

Transfected plasmids do not express in iNeurons.

### Potential solution

• Use plasmids with PGK or EF1alpha promoter. In our hands, plasmids with CMV promoter do not express reliably in iNeurons (step 19).
• Increase the amount of Lipofectamine Stem and/or plasmid DNA. Test different combinations of Lipofectamine Stem volume and plasmid DNA amount to optimize transfection efficiency. Examine iNeurons under a tissue culture microscope to ensure that increased amounts of plasmid DNA / Lipofectamine Stem do not negatively affect iNeuron health (step 19).

## Resource availability

### Lead contact

Further information and requests for resources and reagents should be directed to and will be fulfilled by the lead contact, C. Alexander Boecker (alexander.boecker@med.uni-goettingen.de).

### Technical contact

Technical questions on executing this protocol should be directed to and will be answered by the technical contact, Dan Dou (Dan.Dou@pennmedicine.upenn.edu).

### Materials availability

Unique reagents generated in this study are available from the lead contact with a completed Materials Transfer Agreement. Plasmids generated in the studies demonstrating execution of this protocol have been deposited to Addgene.

### Data and code availability

Data generated in the studies demonstrating execution of this protocol have been deposited in Zenodo repositories.^1^^,^^2^ Any additional information required to reanalyze the reported data is available from the lead contact upon request.

## Acknowledgments

We thank Michael Ward (National Institutes of Health), Bill Skarnes (The Jackson Laboratory), and Erika Lara Flores (National Institutes of Health) for their expertise in utilizing resources from the iPSC Neurodegenerative Disease Initiative (iNDI) and the Live-Cell Imaging Facility of the Max Planck Institute for Multidisciplinary Sciences, Goettingen, Germany, for technical support. We thank Carris Borland for assistance with neuronal cultures; Liz Gallagher, Julia Riley, and Kaya Matson for assistance with image acquisition; Cooper Penner for optimizing our custom KymoTracker analysis software; and Jayne Aiken for valuable discussions and feedback. This work was supported by the National Institutes of Health (1 F31 NS124249–01 and T32-AG-000255 to D.D. and R01 NS060698 to E.L.F.H.), the Michael J. Fox Foundation (MJFF-021130 to C.A.B. and E.L.F.H. and MJFF-15100 and MJFF-019411 to E.L.F.H.), and the Else Kröner-Fresenius-Stiftung (2023_EKEA.91 to C.A.B.).

## Author contributions

Conceptualization, D.D., E.L.F.H., and C.A.B.; methodology, D.D. and C.A.B.; investigation, D.D. and C.A.B.; writing – original draft, D.D. and C.A.B.; writing – review and editing, D.D., E.L.F.H., and C.A.B.; funding acquisition, D.D., E.L.F.H., and C.A.B.; supervision, E.L.F.H. and C.A.B.

## Declaration of interests

The authors declare no competing interests.
